# Clinico-epidemiological profile and diagnostic procedures of pediatric tuberculosis in a tertiary care hospital of western Nepal-a case-series analysis

**DOI:** 10.1186/1471-2431-10-57

**Published:** 2010-08-09

**Authors:** Chandrashekhar T Sreeramareddy, Narayan Ramakrishnareddy, Ravi K Shah, Ramkaji Baniya, Pradipta K Swain

**Affiliations:** 1 Department of Community Medicine, Manipal Teaching Hospital, Manipal College of Medical Sciences, Pokhara, Nepal; 2 Department of Community Medicine, Bangalore Medical College and Research Institute, Bangalore, India; 3 Manipal College of Medical Sciences, Pokhara, Nepal; 4 Department of Pediatrics, Manipal Teaching Hospital, Manipal College of Medical Sciences, Pokhara, Nepal; 5 Department of Community Medicine, Melaka-Manipal Medical College, Melaka, Malaysia; 6 Department of Pediatrics, Melaka-Manipal Medical College, Melaka, Malaysia

## Abstract

**Background:**

Changing epidemiology and diagnostic difficulties of paediatric tuberculosis (TB) are being increasingly reported. Our aim was to describe clinico-epidemiological profile and diagnostic procedures used for paediatric TB.

**Methods:**

A retrospective case-series analysis was carried out in a tertiary care teaching hospital of western Nepal. All pediatric TB (age 0-14 years) patients registered in DOTS clinic during the time period from March, 2003 to July, 2008 were included. Medical case files were reviewed for information on demography, clinical findings, investigations and final diagnosis. Analysis was done on SPSS package. Results were expressed as rates and proportions. Chi square test was used to test for statistical significance.

**Results:**

About 17.2% (162/941) of TB patients were children. Common symptoms were cough, fever and lymph node swelling. The types of TB were pulmonary TB (46.3%, 75/162), followed by extra-pulmonary TB (41.4%, 67/162). Twelve patients (7.4%) had disseminated TB. Distribution of types of TB according to gender was similar. PTB was common in younger age than EPTB which was statistically significant. EPTB was mainly localized to lymph node (38, 50.7%), and abdomen (9, 12%). Five main investigations namely Mantoux test, BCG test, chest radiograph, erythrocyte sedimentation rate (ESR) and fine needle aspiration cytology (FNAC) or biopsy were carried out to diagnose TB.

**Conclusions:**

Paediatric TB in both pulmonary and extrapulmonary forms is a common occurrence in our setting. Age incidence according to type of TB was significant. Diagnosis was based on a combination of epidemiological and clinical suspicion supported by results of various investigations.

## Background

World Health Organization (WHO) reports that about two billion i.e. nearly one third of the world's population is currently infected with mycobacterium tuberculosis. Developing countries account for 95% of the burden of tuberculosis (TB) and 99% of the TB mortality reported worldwide [1]. It is estimated that about 9% of the TB cases globally occur among children less than 15 years of age. The same proportion in low-income countries is 15% [2]. TB among children is important for public health professionals since it is an indicator of the recent transmission of TB in the community. Contact investigations of pediatric TB patients may lead to improved case-finding among adult patients [3]. However, the national TB control programs lay more emphasis on sputum smear-positive adult TB cases since they are highly infectious. As a result childhood TB is often neglected by TB control programs due to the difficulties in confirming diagnosis, over estimating the protective efficacy of BCG vaccine [4]. Moreover, diagnosis of TB among children may be more challenging in resource-poor settings like Nepal. There has been an increasing concern about TB among children who are HIV seropositive [5]. Studies from Taiwan, USA and Saudi Arabia have reported about epidemiology and clinical features childhood TB [6-9]. Clinical presentation may depend on the epidemiological situation of TB and HIV in that country. Diagnostic methods followed for childhood TB may vary depending on the available resources in the health-care setting.

In Nepal, about 45% of the total population is infected with TB and an estimated 20,000 new infectious cases of TB are reported each year. Sentinel surveys have reported that the rate of HIV seropositivity in all age groups has increased from 0.6% in 1995/96 to 2.4% in 2006/07[10]. However, studies on epidemiology, clinical profile and diagnostic methods of childhood TB from low-income countries like Nepal are lacking. Therefore, we carried out this study to describe the clinico-epidemiological profile and diagnostic processes of pediatric TB patients.

## Methods

### Study setting

Manipal Teaching Hospital (MTH) is a tertiary care hospital which is affiliated to Manipal College of Medical Sciences (MCOMS). MTH serves patients from Pokhara city and remote hilly areas of western Nepal. In March 2003, DOTS (Directly Observed Treatment, Short course) clinic was started in MTH as a part of involvement of medical colleges in TB control under the National TB Program (NTP) of Nepal. Guidelines of NTP are followed for diagnosis of TB in all the clinical departments of MTH. TB patients diagnosed in various clinical departments of MTH are referred to DOTS clinic where they are registered and receive treatment according to the guidelines of NTP.

### Data collection

Ethical clearance was obtained from ethical committee of MCOMS and permission to access medical records was obtained from medical superintendent of MTH. All pediatric TB (age 0-14 years) patients registered in DOTS clinic during the time period from March, 2003 to July, 2008 were included for the study. A list of patients with their hospital numbers was obtained from the DOTS clinic and original medical case files were traced from medical records department. Medical case files, reports of chest radiographs and laboratory investigations were reviewed to obtain the necessary information about diagnosis of TB. The information collected included symptoms and their duration, findings of sputum examination or gastric lavage for the presence of acid fast bacilli (AFB), localization of lesions in the chest radiographs, details of laboratory and/or histopathological examination for diagnosis for extra-pulmonary TB. We also gathered information about household contact with an active case of pulmonary TB, tests for HIV infection, history of BCG vaccination and/or presence of BCG scar (at least four millimeters in size), Mantoux test and BCG test/accelerated BCG response (When BCG is given to a child with TB, the reaction occurs at the site of vaccination within 48-72 hours as compared to usual late reaction which occurs after 3-6 weeks in a child without TB. Appearance of a papule or an induration more than 5 mm in size at the test site was considered as positive BCG Test) [11]. For the purpose of our analysis type of TB was classified as isolated pulmonary TB, extra-pulmonary TB, pulmonary TB with extra-pulmonary TB (only one extrapulmonary site) and disseminated (pathology in more than two sites) and miliary TB. Data was entered into SPPS version 13 (Statistical Package for Social Sciences) package. Data was presented as rates and proportions. Statistical significance of difference in proportions was tested using chi square test and a p-value less than 0.05 was considered as significant.

## Results

**Figure 1:**
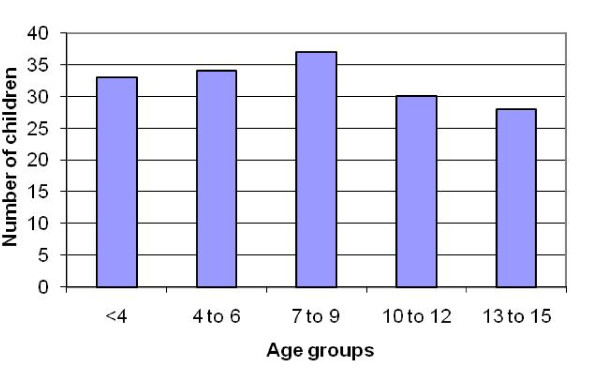
**Age distribution of all TB patients**.

A total 941 TB patients were diagnosed during March 2003 to July 2008. Of these 178 were children aged 14 years or less (i.e. 18.9% of all cases were childhood TB). These cases were diagnosed and referred to DOTS clinic for treatment. From the list of 178 cases obtained from DOTS register, medical case files of 16 patients could not be traced. Median age of the children was 7.5 years (interquartile range, 4 to 12 years). Age of the patients ranged from 6 months to 14 years. Age distribution of TB patients is shown in figure 1 and was similar in all age groups. Male to female ratio was 1:1 with 81 children in each group. The most common presenting symptoms were fever, cough, lymph node swelling and pain as shown in table 1.

**Table 1:** Presenting symptoms of the children diagnosed as Tuberculosis

Complaint	Number (%)*
Fever	70 (43.2)

Cough	42 (25.9)

Lymph node swelling	25 (15.4)

Pain (abdomen, chest etc)	19 (11.7)

Loss/decreased appetite	13 (8.0)

Failure to thrive	11 (6.8)

Breathlessness	7 (4.3)

Discharge from the ear	5 (3.1)

Weight loss	4 (2.5)

Redness in the eye	4 (2.5)

Others	9 (5.6)

* Each patient may have more than one symptom

**Figure 2:**
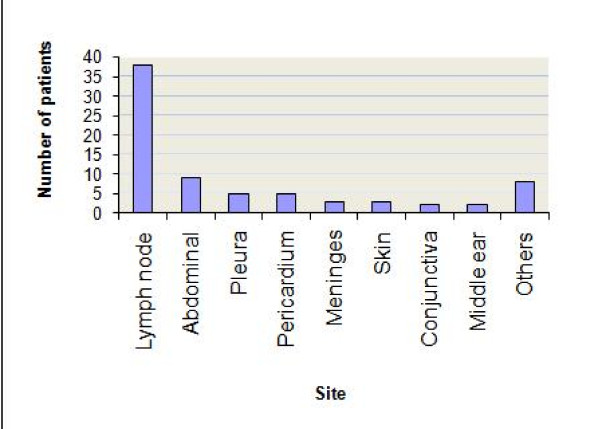
**Sites of extra-pulmonary tuberculosis**.

Most common form of TB was pulmonary TB followed by extra-pulmonary TB. Twelve patients were diagnosed as disseminated TB (one child had miliary TB). Distribution of types of TB according to age groups is shown in table 2. Pulmonary TB was common in younger age group as compared to extra-pulmonary TB which was common in older age group. This difference was statistically significant. Distribution of type of TB among male and female patients (data not shown) was not statistically significant (chi square 3.29, p = 0.35). The sites of extrapulmonary TB are shown in figure 2. Out of 75 patients who were diagnosed as extra-pulmonary TB (inclusive of combined type); most common sites were lymph node, and abdomen. Less common sites of extra-pulmonary TB were pleura, pericardium, and meninges.

**Table 2:** Types of Tuberculosis according to age

Age group (Years)	Type of tuberculosis (Number and percentage)				Total
		
	Isolated Pulmonary	Isolated Extrapulmonary	Combined (PTB + EPTB)	Disseminated/ Miliary	
< 4	26 (34.7)	4 (6.0)	-	3 (25.0)	33 (20.4)

4-6	17 (22.7)	14 (20.9)	1 (12.5)	2 (16.7)	34 (21.4)

7-9	12 (16.0)	20 (29.9)	-	5 (41.7)	37 (22.8)

10-12	10 (13.3)	14 (20.9)	5 (62.5)	1 (8.3)	30 (18.5)

13-15	10 (13.3)	15 (22.4)	2 (25.0)	1 (8.3)	28 (17.3)

Total	75 (100)	67 (100)	8 (100)	12 (100)	162 (100)

Chi square = 36.1, p < 0.0001

Immunisation history for BCG and/or presence of BCG scar was present in 93 (57.4%) patients and absent in 20 (12.3%) patients. For 49 (30.2%) patients information was not available. Out of 162 cases reviewed, HIV spot test was done for 36 (22.2%) patients and was positive in eight children. History of contact with a case of TB was available for only 126 (77.7%) out of 162 patients. For remaining 36 (22.2%) patients data was not available. Of these 126 patients for whom information about history of contact was available, only 38 patients had positive history of contact.

Upon review of the investigations carried out for diagnosis we found that five main investigations namely Mantoux test, BCG test (accelerated BCG response), chest radiograph, ESR and FNAC or biopsy were carried out. Table 3 shows various investigations carried out according to type of TB. Microscopy for AFB was done on samples of sputum, gastric lavage and also used pus, ear swab or cerebrospinal fluid. However, microscopy was positive only in a small proportion (< 20%) of children in both pulmonary and extra-pulmonary TB and none in disseminated TB. Sputum for AFB was not done for 13 of 58 children who were aged above 10 years. Mantoux test was done for 57 children diagnosed as PTB and 66% of them had a positive test (induration of at least 10 millimeter was considered as positive) [12]. Mantoux test was done for 45 children diagnosed as EPTB and 71% of the children were tested positive. However, BCG test was done for only 31 children diagnosed as PTB and for 15 children diagnosed as EPTB. Overall, BCG test was done for 49 patients and was positive in more than 90% of both PTB and EPTB patients. Chest radiograph was positive in 94% (72/76) of PTB patients and 63% (36/57) of EPTB patients. Lymph node TB was mainly diagnosed by FNAC. FNAC was done for 38 children and was positive for TB in 32 of them. For other (abdominal, pleural, pericardial, meningeal) extrapulmonary manifestations of TB, diagnosis was based on positive findings of special investigations like ultrasound (16/18) lumbar puncture (2/2), biopsy of the skin (1/1). Middle ear TB was diagnosed based on presence of AFB in ear swab. Phlyctenular keratoconjunctivtis was diagnosed based on positive Mantoux test and/or positive chest x-ray.

**Table 3:** Diagnostic procedures/investigations carried out

Investigations or diagnostic procedures	Type of tuberculosis (Figures show number positive out of number of patients on whom procedure/investigation done)		
	
	Pulmonary (n = 83)	Extra-pulmonary (n = 75)	Disseminated (n = 12)
Mantoux test	38/57	32/45	9/10

BCG test	29/31	14/15	3/3

Chest radiograph	72/76	36/57	8/9

Elevated ESR	32/55	20/54	7/10

Gastric lavage for AFB	3/27	1/12	0/6

Sputum for AFB	5/36	2/37	0/2

FNAC	-	32/38	-

ultrasound	-	16/18	-

Biopsy	-	7/7	-

lumbar puncture for cerebrospinal fluid analysis	-	2/2	-

## Discussion

In our analysis of medical case files/records we found that childhood TB accounted for nearly a fifth of all TB patients. Common symptoms were fever, cough and lymph node swelling. Though distribution of all forms of TB was similar across all age groups, there was slight peak for pulmonary TB among children aged less than four years and extra-pulmonary TB after 10 years of age. Most common extra-pulmonary site was lymph node (mainly cervical). For the diagnosis of pulmonary TB, chest radiograph, Mantoux and BCG tests were done while extra-pulmonary TB was diagnosed using various diagnostic procedures.

Childhood TB is often neglected in high-burden countries. It is thought that children suffer from TB less often and less severely than the adults [13]. On the contrary, in high-burden countries a considerable proportion of TB patients are children and TB related morbidity and mortality is high among children [14]. Despite the decrease in TB burden since 1960 s there was resurgence during the nineties due to HIV epidemic [15]. The age incidence for all forms TB showed a bimodal distribution in two studies from Taiwan [6,7]. In our analysis, we did not find such pattern but we found two separate peaks for PTB (< 4 years) and EPTB (after 10 years). One possible explanation for such pattern may be higher TB prevalence in Nepal. A higher frequency of exposure to infectious patient at an early age could have resulted in pulmonary disease. It is also possible that BCG vaccination may have protected them from serious extra-pulmonary forms of TB [16]. This is supported by our data that nearly 60% of the children had received BCG vaccine and/or BCG scar was present. Some studies have reported that BCG may protect children from TB infection also [17,18]. However, studies from other countries have reported that young children are more likely to develop extra-pulmonary TB [19,20]. In our analysis, we found that 55% of all TB patients had extra-pulmonary involvement and 10/12 patients of disseminated or miliary TB were younger than 10 years and most of the children who had extra-pulmonary TB were older than 4 years.

Few studies have suggested about increasing trends of extra-pulmonary manifestations among children as well adults [19,20]. Most common extra-pulmonary site in our setting was lymph node similar to studies reported from elsewhere [20-22]. The second common site was gastrointestinal. Increasing trend for extra-pulmonary TB may be attributed to HIV infection. We cannot attribute a higher proportion of EPTB to HIV infection from our data since HIV testing was only done for a fifth of all patients. Routine HIV testing is not done for TB suspects in our setting. Rather, HIV testing is done only on the grounds of clinical and/or epidemiological suspicion. Higher frequency of extra-pulmonary TB could be due to transmission of bovine TB in an agrarian country like Nepal where proximity with livestock is common. This could also be the reason for gastrointestinal system being second common site of extra-pulmonary TB. However, there is a lack of adequate evidence to support this hypothesis.

In our analysis, 12 (7.4%) children had disseminated TB which is a cause for concern. It is noteworthy that most of these children were also younger than 10 years. Non-specific manifestations of pediatric TB, lack of diagnostic facilities and lack of suspicion of TB among physicians in smaller health facilities may be the reasons for presenting to a teaching hospital at later stages of disease. In a large proportion of patient files symptoms were only listed without recording their duration since onset. Therefore, we could not assess if there was a longer time duration since onset of symptoms until diagnosis was made in our hospital.

It is a well known fact that diagnosis of TB in pediatric age group is difficult [23]. Our analysis about the diagnostic procedures revealed that there was no single diagnostic procedure which could be used as a gold standard test. Most children underwent many diagnostic procedures before a final diagnosis of TB was made. There are some notable points to be made about diagnostic procedures followed in our setting. The diagnostic criteria for both forms of TB were not clear in the case files. PTB was diagnosed mainly based on chest radiograph, Mantoux test, BCG test and ESR. Special investigations were done for EPTB patients but diagnostic criteria were not explicit in the case files. However, we read the reports of chest radiographs, ultrasound, biopsies etc to corroborate with the final diagnosis. Chest radiograph was used for both pulmonary and extra-pulmonary though its reliability as diagnostic tool is questionable [24]. A high proportion (94%) of chest radiographs were interpreted as positive for patients diagnosed as PTB. Clinical suspicion and evidence from positive results of other diagnostic tests may have prompted the physicians to interpret chest radiographs as positive for TB. Sputum or other specimens for presence of acid fast bacilli which is gold standard test for adult TB was occasionally used. We emphasize this point since sputum AFB was not done for all children aged above 10 years. Mantoux (skin) test was done on a fairly high proportion of patients with all forms of TB and about two-thirds of those tested were interpreted as positive. There is a difficulty in interpreting a positive Mantoux test in our setting where universal BCG vaccination is done during neonatal period [25]. Though negative Mantoux test does not rule out TB, a positive test may be a useful diagnostic tool in a resource-limited setting like ours. Extrapulmonary TB was diagnosed mainly by FNAC and biopsy. These findings emphasize about the diagnostic difficulties faced by physicians in resource-limited settings. Such findings have been reported from other countries as well [24,26,27].

Our study had a few limitations. Some important clinical and demographic information were incomplete or missing from the medical case records. When demographic information was not available in medical case files, we cross-checked patient's name with hospital number or outpatient registration number and also with DOTS center register to find the information about age and gender. There was no means to find missing clinical data as we did a retrospective analysis. Very important information about weight and/or height was not available for most of the children and was not included for this analysis. With best of our efforts we could not trace a few medical records from the archives.

## Conclusion

Pediatric TB is common in our setting. Common extrapulmonary sites were lymph nodes and gastrointestinal system. Diagnosis of TB was not systematic and mostly based on a combination of epidemiological and clinical suspicion supported by results of various investigations.

## Competing interests

The authors declare that they have no competing interests.

## Authors' contributions

**CTS **contributed to the study design, assisted in the data collection, and drafted first version manuscript for publication. **NRR **Assisted in study design and co-drafted the manuscript for publication. **RKS **reviewed the medical case files, conducted the data analysis and prepared the results. **RB **reviewed the medical case files, conducted the data analysis and prepared the results. **PKS **assisted in study design, prepared the case record form, and revised the earlier versions of the manuscript. All authors read and approved the final manuscript to be submitted for Publication.

## Pre-publication history

The pre-publication history for this paper can be accessed here:

http://www.biomedcentral.com/1471-2431/10/57/prepub
